# A Context-Recognition-Aided PDR Localization Method Based on the Hidden Markov Model

**DOI:** 10.3390/s16122030

**Published:** 2016-11-30

**Authors:** Yi Lu, Dongyan Wei, Qifeng Lai, Wen Li, Hong Yuan

**Affiliations:** 1 Academy of Opto-Electronics, Chinese Academy of Sciences, Beijing 100094, China; luyi_aoe@163.com (Y.L.); laiqifeng@aoe.ac.cn (Q.L.); wen.li@aoe.ac.cn (W.L.); yuanh@aoe.ac.cn (H.Y.); 2 University of Chinese Academy of Sciences, Beijing 100049, China

**Keywords:** PDR, context recognition, HMM, indoor localization, turn detection

## Abstract

Indoor positioning has recently become an important field of interest because global navigation satellite systems (GNSS) are usually unavailable in indoor environments. Pedestrian dead reckoning (PDR) is a promising localization technique for indoor environments since it can be implemented on widely used smartphones equipped with low cost inertial sensors. However, the PDR localization severely suffers from the accumulation of positioning errors, and other external calibration sources should be used. In this paper, a context-recognition-aided PDR localization model is proposed to calibrate PDR. The context is detected by employing particular human actions or characteristic objects and it is matched to the context pre-stored offline in the database to get the pedestrian’s location. The Hidden Markov Model (HMM) and Recursive Viterbi Algorithm are used to do the matching, which reduces the time complexity and saves the storage. In addition, the authors design the turn detection algorithm and take the context of corner as an example to illustrate and verify the proposed model. The experimental results show that the proposed localization method can fix the pedestrian’s starting point quickly and improves the positioning accuracy of PDR by 40.56% at most with perfect stability and robustness at the same time.

## 1. Introduction

Nowadays, with the rapid development of computing technology, the demand for location based services (LBS) is rapidly increasing [1]. Global navigation satellite systems (GNSS) have been successfully used for outdoor scenarios; however, it is difficult to use it indoors because of signal attenuation [2].

Recently, various novel indoor localization techniques have been proposed, such as infrared light, Bluetooth, ultrasound, wireless local area networks (WLAN) [3], micro-electro-mechanical system (MEMS) and cellular network [4]. Among these methods, techniques based on smartphone sensors have attracted much more attention because of the popularity of mobile phones [5]. However, some of them require additional infrastructures such as access points (AP) and base stations. Thus, in this paper, we mainly concentrate on Personal Dead-reckoning (PDR), in which only the inertial sensor of the phone is used [6]. This relative localization method measures and tracks the momentary location and trajectory of a walking person dependently using the smartphone without any external sensors. Unfortunately, PDR’s main problem lies in the fact that the positioning errors will accumulate over time very quickly due to the drift caused by noise, especially for the low-cost and low-performance sensors used in smartphones.

Different solutions can be used to eliminate the positioning errors in PDR: (1) an improved PDR algorithm, which makes the step detection, estimation of stride length and heading more sophisticated [7,8]; (2) systems integrated by external sensors to correct PDR. For the first resolution, Kim proposed in [7] a new reliable step determination method based on pattern recognition from the analysis of the acceleration of the foot during one step of the walking and a stride estimation method by analyzing the relationship between stride, step period and acceleration. Furthermore, its integration method of gyroscope and magnetic compass gave a reliable heading. In [8], Liu et al. took the diverse ways in which people use smartphones into consideration and designed a new gait step detection algorithm to detect steps. The authors also divided the pedestrian walking process into single posture and posture switching processes, and corrected the heading of the two walking processes. Generally speaking, these kind of solutions can improve the performance of PDR radically, and reduce its errors, but they are relative localization methods after all. Without human input or external references, the smartphone can hardly infer its initial position, which is the basis for distance calculation, since all that a smartphone can learn is its acceleration [9]. Moreover, the current researches for the second solution are more extensive. Parts of these studies exploit external sensors to calibrate PDR, such as the fusion of radio frequency identification (RFID) signals and PDR described in [10]. In addition, Zhang applied WLAN signals to supply PDR in [11]. Although these methods correct the cumulative errors in PDR, installation of external sensors is time consuming and expensive, and also needs human labor in the process of preparing the signal fingerprint collection before localization. Other studies [12,13,14,15] have focused on map matching algorithms, which match the trajectory obtained by the localization system and a database of road information related on electronic maps [16]. However, this is very complex and cumbersome, requiring the storage of a large amount of map information which leads to extremely high time complexity [13], because it only uses the trajectory calculated by PDR, regardless of some possible characteristic contexts during the moving process, such as stairs, elevators, ramps, detours, etc. In order to simplify the map matching algorithm, Jiménez et al. used an inertial measurement unit (IMU) sensor fixed on a person’s body to detect the movement of going up a ramp [6], which combines the trajectory with the recognized features, so that saves the storage of map information. After detection, the ramp is checked for association with one of the positions stored in a database. For each associated ramp, a position correction is fed into the PDR system in order to refine the PDR solution. However, this method has to be operated under the condition of a simple environment with ramps of different slopes. In other words, it is unable to find the starting point and an individual’s position in complex environments.

In view of the merits and drawbacks of PDR solutions, we can see that the matching algorithm integrated the characteristic contexts on the map can greatly reduce the storage dimensions compared to traditional map matching algorithms. Furthermore, it can be directly realized through an intelligent terminal without installing external sensors, which saves time, storage, and labor costs.

Therefore, in this paper, we extend the ramp scene of [6] to various contexts, and design a context-recognition-aided PDR localization method. At the same time, a Hidden Markov Model (HMM) is utilized in this method because we find that the output sequences of characteristic contexts satisfy the Markov property. Compared to traditional map matching and fingerprint algorithms, this method needs less information which can be measured directly and adjusted quickly whenever the map changes, and it is more reliable because the geographical features are more stable than Wi-Fi or Bluetooth signals. Our proposal corrects positioning errors after finding the starting point quickly by matching the current pedestrian position to the characteristic context in a pre-stored context database using HMM, so that improves the positioning accuracy and stability.

The remainder of this manuscript is organized as follows: Section 2 presents the structure of the context-recognition-aided PDR localization method based on HMM. Section 3 describes the context recognition methods, especially the details of turn detection. Section 4 explains the HMM matching algorithm. Section 5 shows the results and discussion for several indoor localization experiments. Finally, in last section, we offer the main conclusions drawn from this work.

## 2. The Structure of Context-Recognition-Aided PDR Localization Method Based on HMM

**Figure 1:**
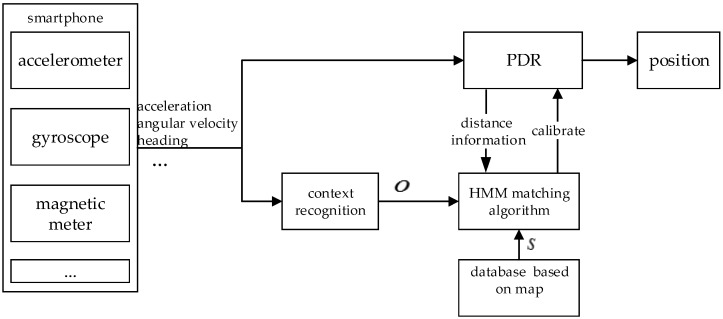
The diagram of the proposed method.

The structure of context-recognition-aided PDR localization method based on HMM is shown in Figure 1, and is mainly divided into three parts: PDR positioning, context recognition and the matching algorithm.

**Figure 2:**
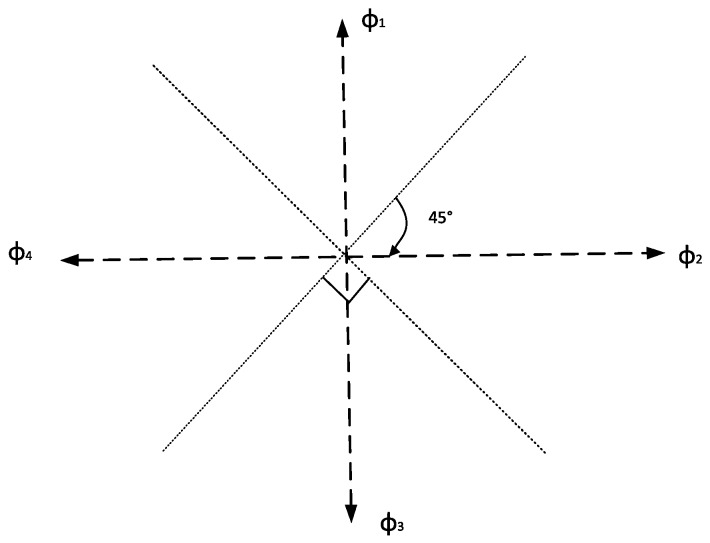
The heading determination graph.

Inertial sensors of smartphones, including accelerometer, gyroscope, and magnetic meter, are used in PDR to estimate the occurrence of steps, stride length and heading [5]. For each step, the user’s position can be predicted by:

(1) $$\{\begin{array}{l} x_{i+1}=x_{i}+d_{i}\cos\left(\theta_{i}\right)\\ y_{i+1}=y_{i}+d_{i}\sin\left(\theta_{i}\right) \end{array}$$

calculated by the nonlinear model proposed in [6] using acceleration measured by the accelerometer. Moreover, θ*_i_* is the heading of this step and considering the low accuracy of smartphone and the impact of various magnetic devices indoors, it is constrained to four directions [φ_1_,φ_2_,φ_3_,φ_4_] using Equation (2) proposed in [17] after the moving direction is measured by the gyroscope and the magnetic meter, as shown in Figure 2.

(2) $$\theta_{i}=\phi_{j}\begin{array}{c} , \end{array}\;\mathrm{if}\;\phi_{j}-45\leq \theta_{i}<\phi_{j}+45\;(1\leq j\leq 4)$$

Based on this, position of the user will be updated after the detection of each step [18]. As mentioned above, an initial position is required at the beginning of the position estimation process, and calibration information is required to reduce error accumulation. In our method, the recognized context’s position pre-stored in the database is employed to calibrate PDR using the HMM matching algorithm.

In this paper, we define a characteristic scene as the context including its type and feature, where type can be corner, stairs, ramp etc. and the corresponding feature is the corner’s orientation θ, the height of the stairs *h* and the orientation of the ramp δ, respectively. It should be noted that the contexts at different position with the same type and feature are considered as the different contexts. As we will show in Section 3, these contexts can be detected using sensors mounted on the smartphone.

For a specific environment, all contexts *s* can be prior surveyed offline and stored in a set $S=\left\{s_{1},\ldots ,s_{k},\ldots ,s_{N}\right\}$, where *N* is the total number of contexts in this environment. The position of every context *s* is pre-stored in a database.

**Figure 3:**
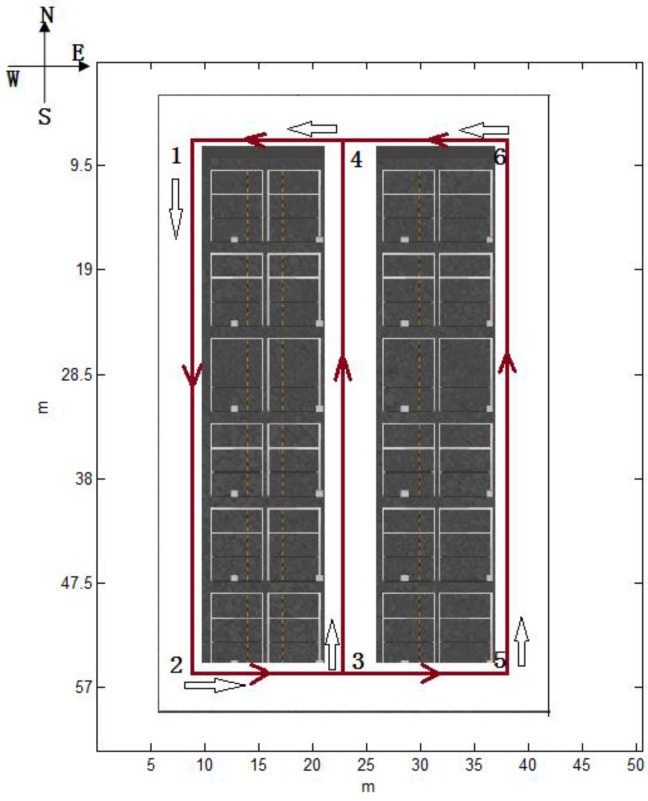
The map of the parking garage at the Beijing New Technology Base of the Chinese Academy of Sciences.

During a practical positioning phase, the contexts a pedestrian passed form a context time series $S=\left(s_{t_{1}^{\prime }},\ldots s_{t_{i}^{\prime }}\ldots s_{t_{P}^{\prime }}\right)$ which is arranged chronologically, where $s_{t_{i}^{\prime }}\in S$ and $t_{i}^{\prime }$ is the time index when the pedestrian passed the context $s_{t_{i}^{\prime }}$. This time series *S* satisfies the Markov property: that is, the current context $s_{t_{i}^{\prime }}$ is independent of all the contexts prior to $s_{t_{i-1}^{\prime }}$. However, as the pedestrian walks, the contexts we detected online form an observed context time series $O=\left(o_{t_{1}}\ldots o_{t_{i}}\ldots o_{t_{Q}}\right)$, where $o_{t_{i}}$ is the detected event containing the type and feature of a context and *t_i_* is the time index when context $o_{t_{i}}$ is recognized. It should be noted that the length of *O* may be different from the length of the theoretical sequence *S* because of misses and false detections during context recognition. For example, we define the orientation of a corner as the pedestrian’s heading after making a turn around a corner. As for the map shown in Figure 3, the black arrows represent corresponding corner’s orientation, if a person walks in accordance with 1-2-3-4 or 1-2-5-6 like the brown line. **S** = {(corner1,south), (corner2,east), (corner3,north), (corner4,west), (corner5,north), (corner6,west)}. If the pedestrian walks along with 1-2-3-4, $S=\left(s_{t_{1}^{\prime }},s_{t_{2}^{\prime }},s_{t_{3}^{\prime }},s_{t_{4}^{\prime }}\right)$ = ((corner1,south), (corner2,east), (corner3,north), corner4,west)), but if corner2 was undetected and other corners’ orientations are detected correctly, $O=\left(o_{t_{1}},o_{t_{2}},o_{t_{3}}\right)=\left(\left(\mathrm{corner},\mathrm{south}),\;(\mathrm{corner},\mathrm{north}),\;(\mathrm{corner},\mathrm{west}\right)\right)$.

If we can match *O* to *S*, we will know the pedestrian’s real trajectory and thus get the position pre-stored in the database, which can be used to calibrate PDR directly. In view of the Markov property of *S*, we use the HMM matching algorithm to match via the joint probability distribution of the sequence, in which the distance information calculated by PDR is also the key information, which will be explained in section 4. In this paper, we will take the corner as the example to illustrate the proposed model and algorithm. To summarize, the context-recognition-aided PDR localization method based on HMM can realize the PDR correction and inhibit the accumulation of positioning errors.

## 3. Context Recognition

As the aided approach in the proposed method, context recognition is the premise of the matching algorithm. There are some previous works in action detection to recognize many different contexts [6,19,20,21], which analyze the signals of an accelerometer placed at different locations on the body to extract some discriminant characteristics of the time-domain or frequency-domain or its distribution by applying wavelet analysis, particle filters and other signal processing methods based on signal features [22]. Similarly, the contexts of stairs, ramps, and elevators can be distinguished by establishing the detection model of height and direction after training, evaluation and analysis using the samples from the pressure meters and the magnetometers in smartphones [6,23,24,25]. For example, the pressure changes obviously and quickly in a short time if a person takes a lift to go up and down, compared to the obvious but slow change when they walk up and down the stairs. Moreover, if the smartphone is mounted on the pedestrian’s foot in the same way shown in [6], steep ramps can be detected by foot’s angle of inclination and the slow change of pressure. In addition, computer vision technology, which detects the pedestrian’s motion image or characteristic objects in the surrounding environment as evidence of human posture or characteristic position [26,27] by extracting the key frames of user’s life videos obtained by the wearable device, is a paramount way for context recognition [28].

In our paper, we present a turn detection method to recognize the context of corners to correct the estimated position of a person. Studies find that the action of turning with respect to walking straight has characteristic features, so the correctness of turn detection is very high. At the same time, corners are common indoors, which is conducive to matching the pedestrian’s current position to a corner in the database.

**Figure 4:**
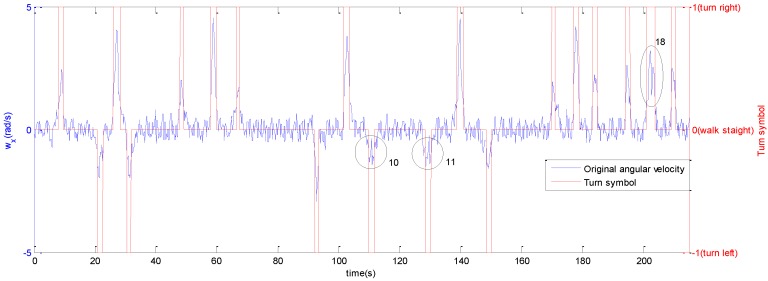
Original angular velocity and the turn symbol based on original data.

When the pedestrian turns, the angular velocity undergoes a severe change compared with the normal walking process whose angular velocity is around 0. A random sample of the original angular velocity of one experimental subject is the blue line shown in Figure 4.

**Figure 5:**
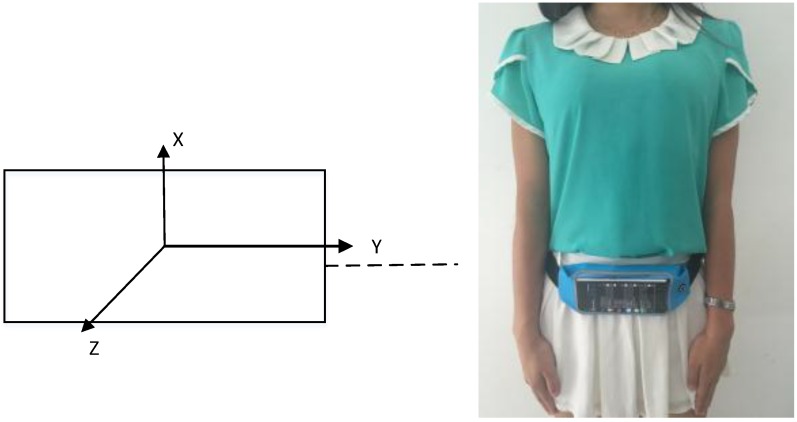
Smartphone’s coordinate system and positioning.

**Figure 6:**
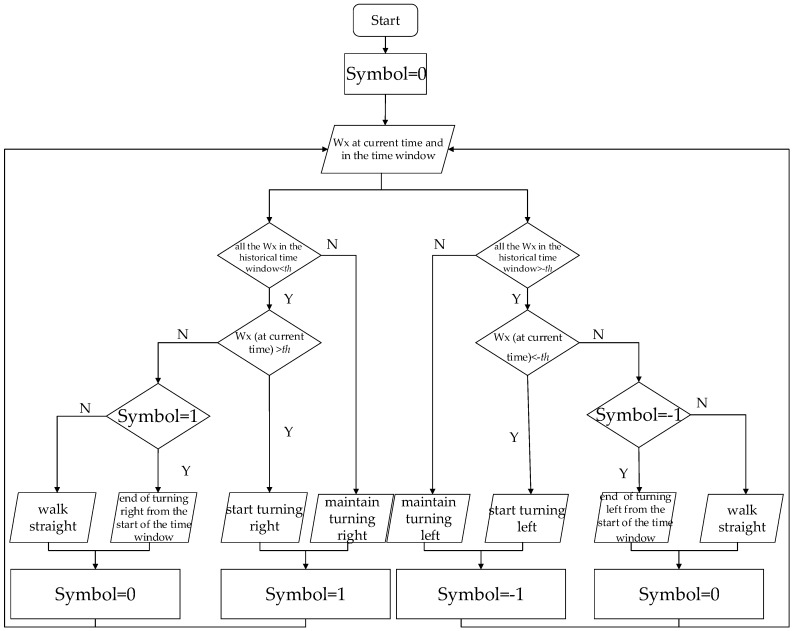
The flow chart of the turn detection algorithm.

However, sometimes the change is not continuous as in the course of turns such as the 10th, 11th and 18th changes which are circled in the figure, so the corners could be recognized using the angular velocity within the time window. Meanwhile, the turn direction (left or right) can be tested depending on the sign (positive or negative) of the angular velocity if the mobile’s position is known. Figure 5 plots a smartphone’s position and its coordinate system, so the angular velocity around the X-axis w_x_ changes violently when turn happens, and w_x_ is negative if turn left, and vice versa. The flow chart of the turn detection is presented in Figure 6.

**Figure 7:**
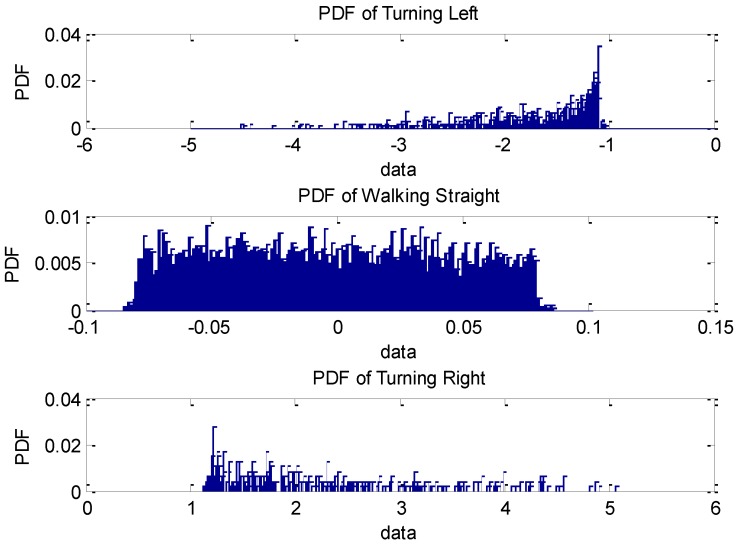
PDF of three movements.

In Figure 6, *th* represents the angular velocity threshold which is a positive number, and “Symbol” stands for the turn symbol during the process of turning, which is 1 if turning right, and −1 if turning left. The choice of *th* is dependent on the statistical analysis of angular velocity collected by the XiaoMi 3 smartphone (XiaoMi, Beijing, China) after we turned 840 times with an angle of ±90° while walking straight. The mean value, variance and probability density function (PDF) are shown in Table 1 and Figure 7. The parameters of the sensors on the XiaoMi 3 are shown in Table 2 [29,30]. It needs to be explained that the orientation sensor measuring the heading is not a real sensor but rather a software sensor which gets its values from combining accelerometer and magnetic field values and applying certain calculations [31].

The result indicates that 1 is a rational threshold for judging turning right and turning left, respectively, because it can detect the turn actions correctly and distinguish them from walking straight. To verify the proposed turn detection scheme, we executed turn tests containing turning left and turning right around a 90°corner during the normal walking process among 10 adults aged 23–25 years old for a total number of 100 tests. The experimental results whose percentage of correct detection is 100% demonstrate that the proposed detection scheme guarantees the recognition of corners. The detected result is the red line in Figure 4. In addition, the heading information after the turn process represents the corner’s orientation. However, there are some limitations of our corner recognition method. For example, the mobile device must be fixed on pedestrian’s body so that the absolute value of the angular velocity when turning is much greater than that while going straight. At the same time, the device’s orientation with respect to the body has to be known, thus the measured heading can be used to match the exact corner in later steps. Secondly, pedestrians are not allowed to turn randomly except for the corners because we assume the pedestrian is located at a corner when a turn action is detected in our algorithm. At last, the time window will lead to the delay of deciding the end of a turn.

Based on this, using the corner recognition algorithm, we can match pedestrian’s location to the corners with the same orientation in the database so as to get user’s position when a turn occurs. However, different contexts may have the same features in complex situations and context recognition error exist, which lead to the mismatches in traditional map matching systems. To solve these problems, this paper puts forward the HMM matching algorithm to match the right context and definitely determine the pedestrian’s position.

## 4. HMM Matching Algorithm

**Figure 8:**
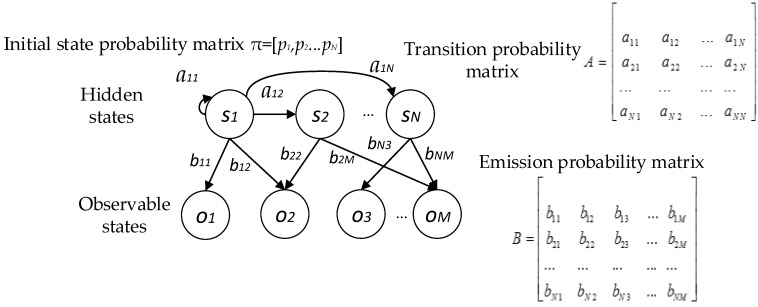
Diagram of HMM.

The matching algorithm based on the Hidden Markov Model (HMM) operates by matching the context information recognized online to the context pre-stored offline in the database. HMM is a statistical model [32], as shown in Figure 8. It is a ubiquitous tool for describing the probability distribution of an observable state sequence $O=\left(o_{t_{1}},o_{t_{2}}\ldots o_{t_{Q}}\right)$ measured by sensors and the hidden state sequence $S=\left(s_{t_{1}^{\prime }},\ldots s_{t_{i}^{\prime }}\ldots s_{t_{P}^{\prime }}\right)$ which cannot be observed directly. Transitions between hidden states are governed by a transition probability $a_{ij}$ in *A*, while the probability of the observable state *o_k_* generated by hidden state *s_j_* can be described by the emission probability *b_jk_* in *B*. The target of HMM matching algorithm is to find out the real sequence of hidden states *S* given the sequence of observable states *O*.

### 4.1. The HMM Matching Algorithm Model

The HMM matching algorithm model can be described with five elements [33], λ = [**S**,**O**,π,*A*,*B*], which include two state sets and three probability matrixes. In this subsection, we take the corner as the example to illustrate the specific implication of these elements in the proposed method.

1. Hidden state set **S**

**Figure 9:**
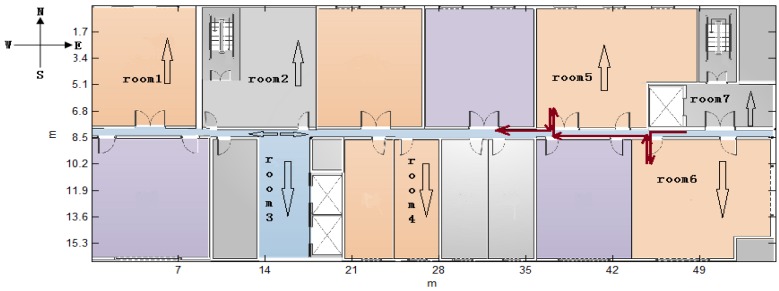
The map of the 8th floor in the main building of the Academy of Opto-Electronics.

In this method, hidden state set $S=\left\{s_{1},s_{2}\ldots s_{N}\right\}$ consists of all contexts in a known environment. The hidden state *s* represents a certain true context, and they form the hidden state sequence $S=\left(s_{t_{1}^{\prime }},\ldots s_{t_{i}^{\prime }}\ldots s_{t_{P}^{\prime }}\right)$ which satisfies the Markov property. If the context is a corner, *s* indicates the exact corner or the room which can be simplified as a corner, and its orientation. For example, in Figure 3 and Figure 9, the hidden state set is {(corner1,south), (corner2,east), (corner3,north), (corner4,west), (corner5,north), (corner6,west)} and {(room1,north), (room2,north), (room3,south), (room4,south), (room5,north), (room6,south), (room7,north)} respectively.

2. Observable state set **O**

The observed state set $O=\left\{o_{1},o_{2}\ldots o_{M}\right\}$ is composed of all possible observed state *o* and *o* is a context detection event that can be observed directly. Taking the corner as an example, *o* includes the detected corner and its orientation. The orientation of a corner has been illustrated in Section 2. Meanwhile, we define the orientation of the room as the heading after the pedestrian enters this room and the corridor’s orientation may be east or west, according to the various walking routes by definition, as shown in Figure 9. Thus, the measured heading can decide whether the pedestrian is walking into or out of a room if we ignore one’s turn actions in the room. From the above definition, a subset of corners can be matched by each turn action and its measured information.

3. Initial state probability matrix π

π expresses the probability distribution of hidden states at the initial time *t*_1_. Supposing that the hidden state set is $S=\left\{s_{1},\ldots ,s_{k},\ldots ,s_{N}\right\}$, π can be described as $\pi =[p_{1},\ldots ,p_{k},\ldots ,p_{N}]$, in which $p_{i}\geq 0$ and $\sum_{i=1}^{N}p_{i}=1$. In the case of unknown starting point, the probability of every context is equal, i.e.,

(3) $$\pi =[\underset{N}{\underset{︸}{\frac{1}{N},\frac{1}{N}\ldots ,\frac{1}{N}}}]$$

4. Transition probability matrix *A*

*A* shows the transition probabilities among any two hidden states in HMM, where $a_{ij}=P(s_{j}|s_{i})$ means the probability that the state is *s_j_* at time *t_j_* under the condition of the state being *s_i_* at time *t_i_* *(i < j)*. The transition probability satisfies $a_{ij}\geq 0$ and $\sum_{j=1}^{N}a_{ij}=1,\;1\leq i\leq N$.

In theory, the transition probability matrix is *A* shown as below if the map is Figure 3.

(4) $$A=\left[\begin{array}{cccccc} 0 & 1 & 0 & 0 & 0 & 0\\ 0 & 0 & 1/2 & 0 & 1/2 & 0\\ 0 & 0 & 0 & 1 & 0 & 0\\ 1 & 0 & 0 & 0 & 0 & 0\\ 0 & 0 & 0 & 0 & 0 & 1\\ 1 & 0 & 0 & 0 & 0 & 0 \end{array}\right]$$

In practice, however, we should take into account of the corner recognition error to determine $a_{ij}$. False detection of turn occurs easily in the vicinity of the angular velocity threshold which is measured by the gyroscope. Thus, the undetected rate of the turn is:

(5) α=Δω

where Δω is the maximal error of gyroscopes measured by large number of statistical experiments. In this paper, we ignore the false alarms while walking on the straight paths, because the angular velocity is far less than the threshold in these cases.

Afterwards, the relationship of any two hidden states can be expressed by three types: single-hop, multi-hop and self-hop. Single-hop means that the theoretical transition probability of two hidden states *s_i_* and *s_j_* is not 0, like 1-2 or 2-3 in Figure 3. Multi-hop indicates the miss detection must happen between two hidden states like 1-3 or 2-6, and self-hop means the hidden states are same at two continuous time point like 1-1 or 2-2. So, the practical transition probability is divided into three conditions. The single-hop’s transition probability is:

(6) $$a_{ij}=a_{ij}\times (1-\alpha )$$

For the multi-hop, we assume the number of undetected corner is *g*, so its transition probability is:

(7) $$a_{ij}=\alpha^{g}\times (1-\alpha )$$

Then, the self-hop’s transition probability is:

(8) $$a_{ii}=1-\sum_{j\neq i}a_{ij}$$

5. Emission probability matrix *B*

Emission probability *b_jk_*, which indicates the probability that the hidden state *s_j_* performs as the observable state *o_k_*, satisfies *b_jk_* ≥ 0. When we calculate *B*, it is necessary to consider with the probability distribution of the measurement errors. Next, we take the corner recognition for example to explain the computation of *B*.

If we assume that the maximal error of heading is Δθ, it is obvious that the corner’s orientation may be constrained wrongly when $\left(\phi_{i}+45^{{}^{\circ}}-\Delta \theta )\leq \theta \leq (\phi_{i}+45^{{}^{\circ}}\right)$ and $\left(\phi_{i}-45^{{}^{\circ}})\leq \theta \leq (\phi_{i}-45^{{}^{\circ}}+\Delta \theta \right)$ according to the PDR heading optimization algorithm mentioned in Section 2.

Therefore, the orientation’s error rate is:

(9) $$\beta =\frac{\Delta \theta \times 2}{90}$$

If the contexts *s_j_* pre-stored in the database have the same orientation with the context *o_k_* recognized online, *b_jk_* is:

(10) $$b_{jk}=(1-\beta )$$

Conversely, if their orientation is different, *b_jk_* is:

(11) $$b_{jk}=\beta$$

For example, in Figure 3, if $\left\{o_{1},o_{2},o_{3},o_{4}\right\}$ = {(corner1,north) (corner1,south), (corner1,west), (corner1,east)}, the emission probability matrix *B* connecting hidden states $\left\{s_{1},s_{2},s_{3},s_{4},s_{5},s_{6}\right\}$ = (corner1,south), (corner2,east), (corner3,north), (corner4,west), (corner5,north), (corner6,west)} is:

(12) $$B=\left[\begin{array}{cccccc} \beta & \beta & 1-\beta & \beta & 1-\beta & \beta \\ 1-\beta & \beta & \beta & \beta & \beta & \beta \\ \beta & \beta & \beta & 1-\beta & \beta & 1-\beta \\ \beta & 1-\beta & \beta & \beta & \beta & \beta \end{array}\right]$$

Then, we can move on to the matching algorithm by the five elements mentioned above.

### 4.2. Matching Procedure Based on HMM

The basic problem solved by HMM matching algorithm is to determine the optimal hidden state sequence $S_{*}=\left(s_{{*}_{t_{1}}}\ldots s_{{*}_{t_{i}}}\ldots s_{{*}_{t_{Q}}}\right)$, according to a specific measured observable state sequence $O_{m}=\left(o_{m_{t_{1}}}\ldots o_{m_{t_{i}}}\ldots o_{m_{t_{Q}}}\right)$ with certain HMM parameters *λ*, where *t_i_* is the time index when the context $o_{m_{t_{i}}}$ is recognized. $S_{*}$ has the largest probability among all possible sequence $\left\{S_{1},\ldots ,S_{e},\ldots \right\}$, where $S_{e}=\left(s_{e_{t_{1}}}\ldots s_{e_{t_{i}}}\ldots s_{e_{t_{Q}}}\right)$ is composed by random permutation using the contexts from **S** in the database:

(13) $$S_{*}=\underset{S_{e}}{\mathrm{argmax}}\;P[S_{e},O|\lambda ]$$

The length of $S_{*}$ may be shorter than the length of real hidden state sequence $S=\left(s_{t_{1}^{\prime }}\ldots s_{t_{i}^{\prime }}\ldots s_{t_{P}^{\prime }}\right)$ because of miss detection of some contexts, but $S_{*}$ can represent *S* to some extent, where $t_{i}^{\prime }$ is the time index the pedestrian passed the context $s_{t_{i}^{\prime }}$. In addition, the distance between any two continuous observed states is also the key information that can be used in our proposed model as shown in Figure 1. We model cumulative errors of PDR, which is represented by $d_{i}-d_{true_{i}}(i>1)$, using a Gaussian distribution *N*(0, σ^2^), where *d_i_* is the distance between $o_{m_{t_{i-1}}}$ and $o_{m_{t_{i}}}$ calculated by PDR and $d_{true_{i}}$ is true distance between hidden states $s_{e_{t_{i-1}}}$ and $s_{e_{t_{i}}}$ pre-stored in the database.

Consequently,

(14) $$\begin{array}{cc} P(S_{e},O_{m}|\lambda ) & =P(O_{m}|S_{e},\lambda )\times P(S_{e}|\lambda )\\ & =\prod_{i=1}^{Q}P(o_{m_{t_{i}}}|s_{e_{t_{i}}},\lambda )\times P(S_{e}|\lambda )\\ & =\pi (s_{e_{t_{1}}})\times (a_{e_{t_{1}}e_{t_{2}}}a_{e_{t_{2}}e_{t_{3}}}\ldots a_{e_{t_{Q-1}}e_{t_{Q}}})\times (b_{e_{t_{1}}m_{t_{1}}}\ldots b_{e_{t_{Q}}m_{t_{Q}}})\times \prod_{i=2}^{Q}N(d_{i}-d_{true_{i}}) \end{array}$$

In this way, the optimal sequence $S_{*}$ can be obtained by the five elements defined in Section 4.1. In our paper, we use the Viterbi algorithm to solve this issue [34,35]. At present, the Viterbi algorithm has two types: method of exhaustion and recursive algorithm [36]. Considering the high efficiency of the recursive algorithm mentioned in [37], we use the recursive algorithm in the proposed method.

At *t_1_*, for any possible hidden state $s_{e_{t_{1}}}$

(15) $$P(s_{e_{t_{1}}},o_{m_{t_{1}}}|\lambda )=\pi (s_{e_{t_{1}}})\times b_{e_{t_{1}}m_{t_{1}}}$$

where $s_{e_{t_{1}}}\in S,\;1\leq e_{t_{1}}\leq N$.

**Figure 10:**
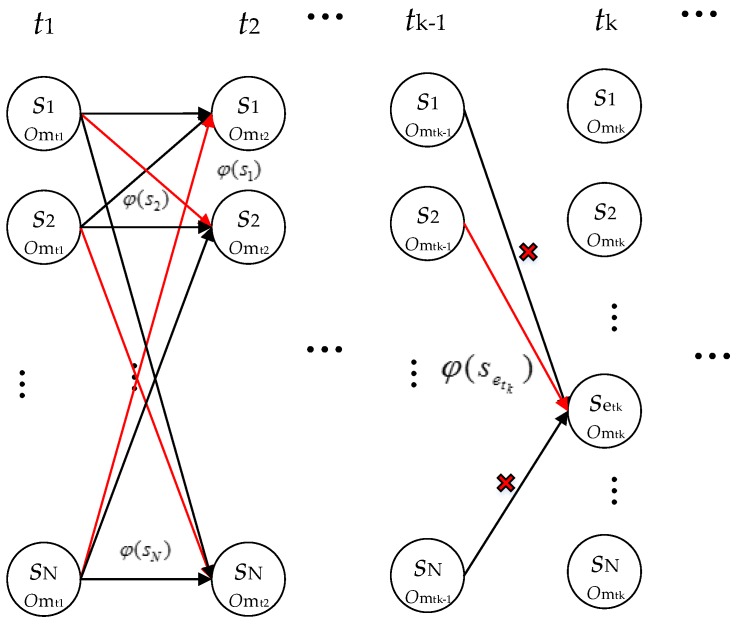
The graph of the Recursive Viterbi algorithm.

From *t_2_*, the recursive algorithm only needs to find the sequence $S_{e_{t_{k}}*}$ with largest probability among all sequences $S_{e_{t_{k}}}$ with the destination $s_{e_{t_{k}}}$ at time *t_k_*, as the red line shown in Figure 10, where $s_{e_{t_{k}}}\in S,\;1\leq e_{t_{k}}\leq N$.

(16) $$\{\begin{array}{c} P(S_{e_{t_{2}}*},O|\lambda )=\underset{1\leq e_{t_{1}}\leq N}{\max}\;P([s_{e_{t_{1}}},s_{e_{t_{2}}}],[o_{m_{t_{1}}},o_{m_{t_{2}}}]|\lambda )\;\begin{array}{c} k=2 \end{array}\\ P(S_{e_{t_{k}}*},O|\lambda )=\underset{1\leq e_{t_{k-1}}\leq N}{\max}\;P([s_{e_{t_{1}}},\ldots s_{e_{t_{k-1}}},s_{e_{t_{k}}}],[o_{m_{t_{1}}},\ldots o_{m_{t_{k-1}}},o_{m_{t_{k}}}]|\lambda )\;\begin{array}{c} k>2 \end{array} \end{array}$$

The probability $P(S_{e_{t_{k}}*},O|\lambda )$ is called the partial probability selected among probabilities calculated by Equation (14) and the optimal path reaches $s_{e_{t_{k}}}$ is named as the optimal route pointer $\varphi (s_{e_{t_{k}}})$ [33]:

(17) $$\varphi (s_{e_{t_{k}}})=S_{e_{t_{k}}*}=\underset{S_{e_{t_{k}}}}{argmax}\;P(S_{e_{t_{k}}},O|\lambda )$$

Therefore, from *t_2_*, once recognizing a context, we will obtain the *N* most possible sequences $\varphi (s_{e_{t_{k}}}),\;1\leq e_{t_{k}}\leq N$, since the total number of hidden states is *N*. In this way, the algorithm is not restarted every time a measurement is received. Conversely, the paths and their associated probabilities from a previous iteration serve as input to the current iteration, along with the new measurements [37].

Last but not least, we need to verify which sequence is the pedestrian’s real track $S_{*}$ among these *N* sequences $\left\{\varphi (s_{1})\ldots \varphi (s_{N})\right\}$. We sort the probabilities $P(S_{e_{t_{k}}*},O|\lambda )$ in descending order, and pick the top two probabilities *P*_max1_ and *P*_max2_ as the candidate matching result. The metric for matching successfully is that the distinguished ratio *P*_max1_/*P*_max2_ has to exceed the threshold *th_p_*. If it is unable to reach the threshold, known as mismatch, it indicates that the result has a high probability of being false, so we do not determine the matching context and retain the current probabilities $P(S_{e_{t_{k}}*},O|\lambda )$ and paths $\varphi (s_{e_{t_{k}}})$, waiting for the next recognition of contexts to recalculate. In contrast, if the result satisfies the metric, we suggest that the matching context is credible, and the position pre-stored in database can be used as the reference to get the start position or calibrate PDR. Therefore, the determination of the threshold is very important because it is closely related to the accuracy of the result, which will be discussed in Section 5.

To sum up, the HMM matching algorithm can rectify accumulated errors in PDR on the basis of inferring the start position by the matching context after context recognition using the sensors’ data collected by the smartphone, so long as the database is known.

## 5. Experiments and Discussion

In this section, we conducted experiments to evaluate the proposed context-recognition-aided localization method based on HMM. The remainder of this section is organized as follows: Section 5.1 tests the reasonable value of output threshold *th_p_* mentioned in Section 4 which is an important parameter that guarantee all the experiments go smoothly. Section 5.2 verifies that the proposed model can determine the start point and Section 5.3 shows the improvement of positioning accuracy of the proposed model. Finally, in last subsection, we analyze the robustness of the model.

The following performance results are based on the data collected from a XiaoMi 3 smartphone mounted on the experimenter’s waist, as shown in Figure 5. Nearly 60 tests were performed by three students (with heights of 1.65 m, 1.7 m, 1.73 m) walking at a normal speed (1.1 m/s, 1.2 m/s, 1.2 m/s on average, respectively) in two environments. One is part of the parking garage with six corners in the Beijing New Technology Base of the Chinese Academy of Sciences, which is approximately a 35.07 m × 57.62 m area, as shown in Figure 3. The other is a test environment with seven available rooms on the 8th floor, which has an area of 54.95 m × 16.8 m, located in the main building of our workplace, Academy of Opto-Electronics, Chinese Academy of Sciences, as shown in Figure 9. In the parking garage, the experimenters walked straight along the path and do not turn, unless they wanted to turn around the corner as indicated by the brown line in Figure 3. And as for the experiments on the 8th floor, we walked straight along the corridor and turned 90° towards to the door when we wanted to enter a room. After taking a few steps following the room’s orientation, we turned 180° and walked for several steps before turning 90° to guarantee that we are walking along the corridor again. In Figure 9, the brown line shows the trajectory if the pedestrian’s route is room6-room5.

### 5.1. Determination of Threshold in HMM Algorithm

The threshold *th_p_* in the HMM algorithm is an essential parameter that determines if the matched context is the right result. Instinctively, some sequences’ probabilities may be larger than the probability of the real sequence, if their contexts or paths’ features are similar. Therefore, *th_p_* should not be too small to avoiding misjudgments. On the contrary, if *th_p_* is too large, *P*_max1_/*P*_max2_ cannot exceed to *th_p_* resulting in a mismatch, even if *P*_max1_ is the probability of the right result. Therefore, we need to testify this impact and select a rational value of *th_p_*, for the purpose of ensuring the correct and fast determination of the matching context.

**Figure 11:**
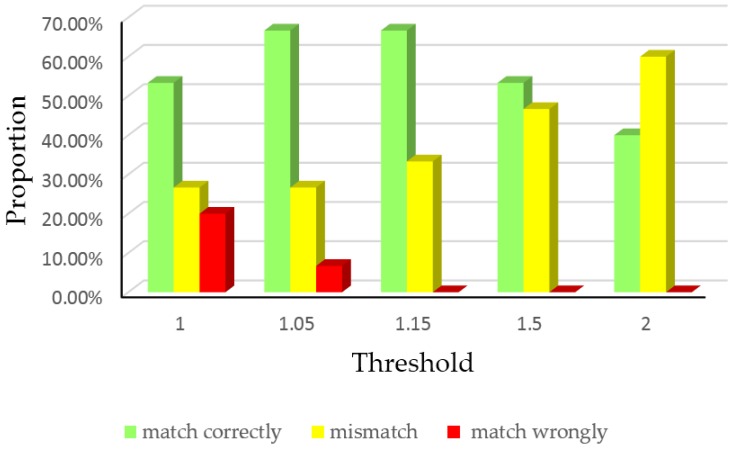
The matching results of different thresholds with the limitation of two contexts.

First, the three students mentioned above did 15 experiments to test the correctness of different thresholds. We turned around just two corners during a normal walk in the parking garage performed seven times, whose routes are 1-2, 2-3, 2-5, 3-4, 4-1, 5-6 and 6-1. For the 8th floor, we only entered two rooms eight times, whose routes are room7-room6, room7-room5, room7-room4, room7-room3, room6-room5, room6-room4 room5-room4 and room5-room3. Figure 11 presents the matching results, where matching correctly means that the matched result is the correct corner or room where pedestrians passed; mismatch means no result outputs because the ratio *P*_max1_/*P*_max2_ is not greater than *th_p_*; matching wrongly means the matched corner or room is wrong.

The histogram manifests that the wrong matching matter occurs if the threshold is relatively small because of the similarity of some paths. When the threshold value grows, the rate of matching wrongly reduces and the mismatch rate rises correspondingly because the probability ratio *P*_max1_/*P*_max2_ cannot reach that high. This result confirms the impact of *th_p_* mentioned at the start of this subsection.

**Figure 12:**
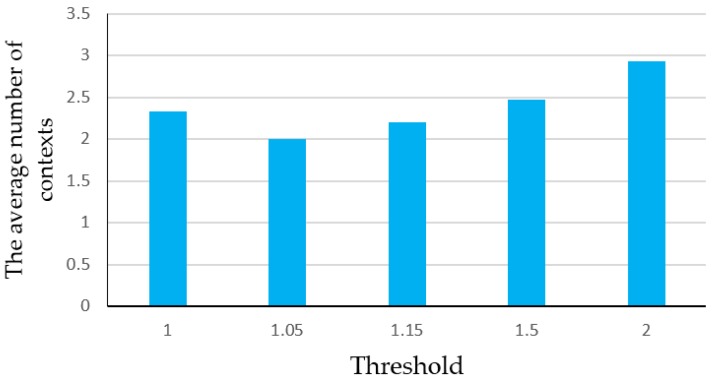
The average number of needed contexts with different thresholds.

In practice, the number of contexts is not limited, so we recorded another 15 experiments performed by three people who walked at a constant speed in two environments respectively and every experiment involved four corners (1-2-3-4, 2-3-4-1, 2-5-6-1, 3-4-1-2, 4-1-2-3, 4-1-2-5, 5-6-1-2, 6-1-2-3) or four rooms (room7-room6-room5-room4, room7-room5-room4-room3, room7-room4- room3-room2, room6-room5-room4-room3, room6-room4-room3-room2, room6-room3-room2- room1, room5-room3-room2-room1). We recorded the number of contexts which experimenters passed by until the result satisfies the output threshold, regardless of its correctness, i.e., the probabilities ratio of two candidate matching results *P*_max1_/*P*_max2_ is larger than *th_p_*. The average number of contexts is shown in Figure 12.

We can see that the number of contexts may be larger than 2, because when a mismatch happens, the HMM matching algorithm retains the past paths and probabilities as the feasible paths and the initial probabilities for the next calculation. Although the selection of 1.05 needs the least number of contexts, it has the probability of matching wrongly as shown in Figure 11, which affects the positioning accuracy significantly. Therefore, 1.15 is appropriate to ensure the matching correctness on the basis of few numbers of contexts, so it should be chosen as the ratio threshold in HMM algorithm in the following experiments.

### 5.2. Determination of the Starting Point

PDR invariably assumes a known initial position, so it is not an independent system and has to be used along with external sensors. Our method, however, can quickly seek the starting position of pedestrians by identifying several contexts in the case of unknown origin.

In this subsection, we conducted 15 tests in the two environments, respectively, in the same way as the second set of experiments described in Section 5.1. Besides the routes mentioned above, we added eight routes (room7-room6-room5-room3, room7-room6-room5-room2, room7- room6-room5-room1, room7-room5-room4-room2, room6-room5-room4-room2, room6-room5- room3-room2, room6-room4-room3-room1, room5-room4-room3-room2) on the 8th floor and one route (6-1-2-5) in the parking garage where some routes were walked twice. Our mission is to see the correctness of the matching result and how long it takes before recognizing the starting point. The results are shown in Table 3, in which ‘Average Number of Contexts’ means the number of contexts we passed by until the result outputs and it can represent the required time before we find the starting point.

From the table, the HMM algorithm can get the starting position precisely in all experiments and requires at least two contexts. Moreover, the average number of contexts shows that users can find the starting point in garage faster than the 8th floor, because the distance between every two contexts differs greatly in parking garage compared with the 8th floor arranged by rooms compactly. This kind of difference of distance results in the severe difference of probabilities of all possible sequences calculated by Equations (14) and (16), so it can decide the result more quickly. In a word, the proposed method based on HMM is a quick and effective way to find the starting point, whose matching accuracy and efficiency is high.

### 5.3. Localization Accuracy

**Figure 13:**
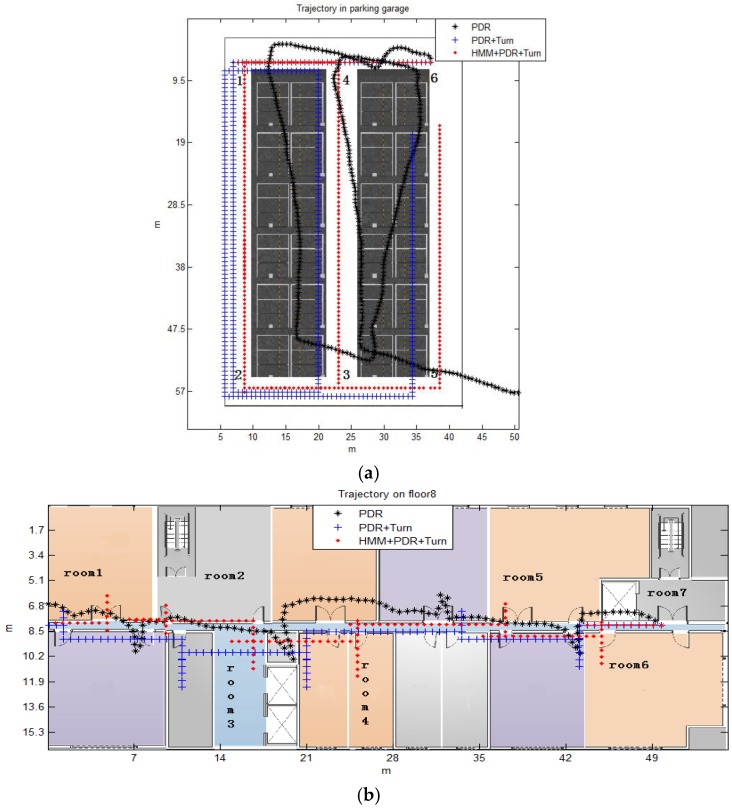
The trajectories of different algorithm. (**a**) In parking garage; (**b**) on the 8th floor.

**Figure 14:**
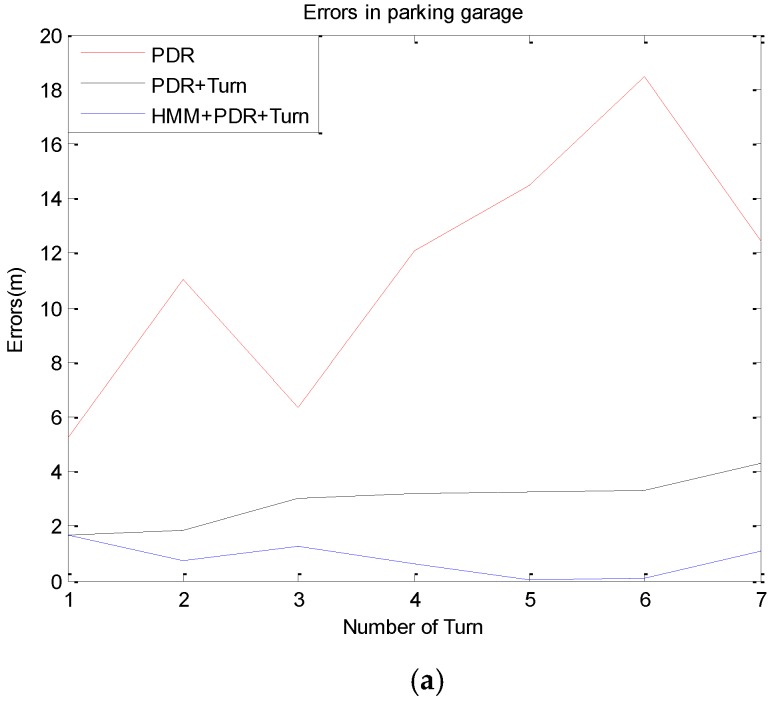

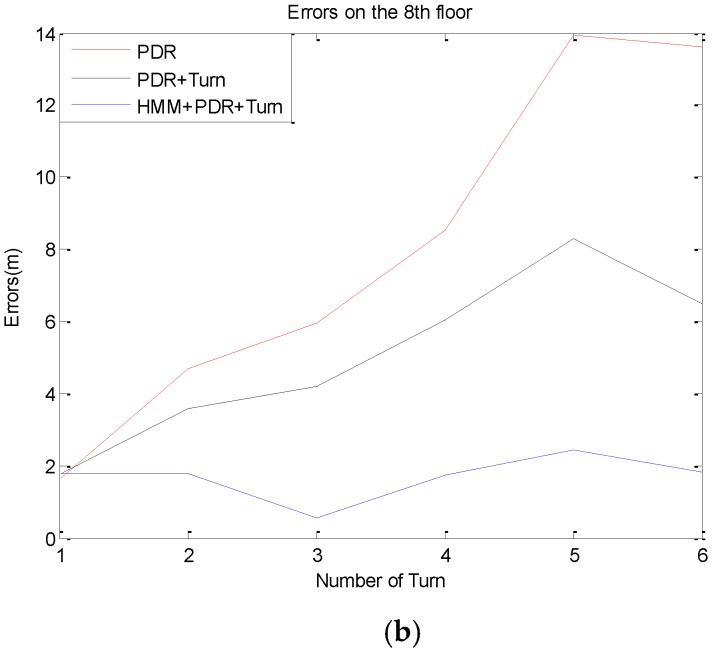
The comparison of positioning errors by different methods. (**a**) In parking garage; (**b**) on the 8th floor.

In further moving processes after determining the starting position, the proposed method corrects the PDR’s positioning errors using the matched context. The performance results given in this subsection are based on data-collection experiments that account for a total of 291 meter-long and 67.8 meter-long trajectories in the garage and 8th floor, respectively. Three students walked at a normal speed (1.1 m/s, 1.2 m/s, 1.2 m/s on average, respectively) for a total of 13 times. The route in the parking garage is 6-1-2-3-4-1-2-5, and it is room7-room6-room5-room4-room3-room2-room1 on the 8th floor of the main building. Here, we compare the positioning errors of three schemes: PDR, PDR + Turn and HMM + PDR + Turn. PDR means the location is obtained by PDR method using the step detection and stride length estimation algorithm mentioned in section 2, but the orientation is the original data collected from smartphone directly without optimization by Equation (2). PDR + Turn optimizes the heading between two recognized corners to a unitary angle and calculated the pedestrian’s position by Equations (1) and (2). HMM + PDR + Turn refers to the context-recognition-aided PDR localization method based on HMM we designed. The target of the comparison is to reveal the contribution of proposed model in the aspect of improving positioning accuracy. The trajectories and errors of one experiment walked by a student whose speed is 1.1 m/s are shown in Figure 13 and Figure 14.

The results suggest that the PDR totally missed the track because of the accumulated errors caused by the sensors’ noise in the smartphone. After combining turn detection with PDR, the trajectory was more regular after adjustment of the angles, but a bias relative to the correct route was obvious sometimes. However, when the HMM algorithms were activated to use the matching context, we obtained almost a perfect elimination of the accumulative positioning errors. The positioning accuracy of the proposed method improved 40.56% at most, and remained stable.

### 5.4. Robustness of the Method

Though our experimental data show a very low false rate for turn recognition, we wanted to examine the robustness of the method when missed detections happen. In this subsection, we used the real data collected in the 13 experiments of Section 5.3 and simulated the failure of context recognition by randomly removing the first or the second detected event artificially from the event stream for testing its fault tolerance. In other words, we set the symbol (1 or −1) of one recognized corner mentioned in Section 3 to 0, which is the symbol for walking straight.

Table 4 compares the result of different false rates. We can see that despite the missed recognition of one context, it still maintains the high correctness, but requires more contexts to obtain the result, in which the miss recognition of the second context needed more time.

**Figure 15:**
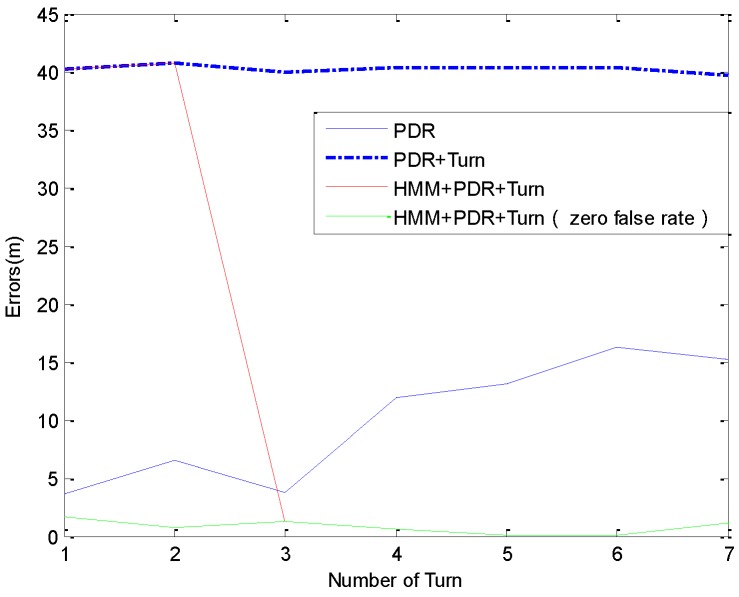
The comparison of positioning errors with different false rate.

Figure 15 shows positioning errors of a simulated experiment, where the second recognized context is removed from the real data of the experiment shown in Figure 13a. We can see that the PDR is not affected by the context recognition. In PDR + Turn method, the heading between two recognized corners is constrained to a unitary angle based on PDR, so it causes the enormous errors when the missed context recognition happens. The errors using HMM + PDR + Turn are large before the correct matching because it uses the same heading optimization algorithm as PDR + Turn, but it is corrected immediately once the matching is successful, and keeps the same accuracy and stability with the method in the condition of zero false rate, no longer affected by the missed detection before.

To sum up, the context-recognition-aided PDR localization method based on HMM has the advantage of good robustness.

## 6. Conclusions

In indoor environments, PDR based on the smartphone cannot realize localization with high precision continuously and stably. In this paper, we design a matching localization model based on characteristic context which can be realized by electronic devices such as smartphones. We match the context information recognized online to the context pre-stored offline in the database and thus get the pedestrian’s location. Compared to traditional map matching and fingerprint algorithms, this method needs less information which can be measured directly and adjusted quickly whenever the map changes, and it is more reliable because the geographical features are more stable than Wi-Fi or Bluetooth signals. In the proposed method, the Recursive Viterbi Algorithm is used to solve the right context sequence, which reduces the time complexity and saves on storage. In the experiment, we detect corners using our proposed detection method and take it as the example to validate the proposed model. The experimental results show that the proposed method can make up for the defects of the PDR individually, which determines the starting position correctly after recognizing a few contexts and compensates the drift of PDR using the matching context. Its positioning accuracy is greatly improved by 40.56% at most, with superior stability and robustness. In the future, we will research various available contexts and the PDR method based on devices with arbitrary posture.

## Figures and Tables

**Table 1:** The statistical result of angular velocity of three movements.

Movement	Mean/Rad·s^−1^	Variance/Rad·s^−1^
Turn left	−1.7425	0.4242
Turn right	2.2055	0.9155
Walk straight	−0.0019	0.0021

**Table 2:** The parameters of the sensors on the XiaoMi 3 smartphone.

Parameter	Accelerometer	Gyroscope	Magnetic Meter
Model	MPU-6050	MPU-6050	AK8963
Manufacturer	InvenSense	InvenSense	AKM
Measurement	acceleration	angular velocity	magnetic field
Range	±20 m/s^2^	±35 rad/s	0–9830 μT
Accuracy	1.5 × 10^−1^ m/s^2^	3 × 10^−3^ rad/s	3 μT

**Table 3:** The results of finding the starting point.

Scene	Correctness/%	Average Number of Contexts
garage	100	2
floor 8	100	2.067

**Table 4:** The results with different false rate.

Scene	Correctness/%	Average Number of Contexts
Zero false rate	100	2
One missed detection	92.3	3.917
